# Integrating Deep Learning with Electronic Health Records for Early Glaucoma Detection: A Multi-Dimensional Machine Learning Approach

**DOI:** 10.3390/bioengineering11060577

**Published:** 2024-06-07

**Authors:** Alireza Karimi, Ansel Stanik, Cooper Kozitza, Aiyin Chen

**Affiliations:** 1 Department of Ophthalmology, Casey Eye Institute, Oregon Health and Science University, Portland, OR 97239, USA; anselstanik@gmail.com (A.S.); cooperkozitza@gmail.com (C.K.); chenai@ohsu.edu (A.C.); 2 Department of Biomedical Engineering, Oregon Health and Science University, Portland, OR 97239, USA

**Keywords:** glaucoma, early detection, machine learning algorithms, clinical data

## Abstract

Background: Recent advancements in deep learning have significantly impacted ophthalmology, especially in glaucoma, a leading cause of irreversible blindness worldwide. In this study, we developed a reliable predictive model for glaucoma detection using deep learning models based on clinical data, social and behavior risk factor, and demographic data from 1652 participants, split evenly between 826 control subjects and 826 glaucoma patients. Methods: We extracted structural data from control and glaucoma patients’ electronic health records (EHR). Three distinct machine learning classifiers, the Random Forest and Gradient Boosting algorithms, as well as the Sequential model from the Keras library of TensorFlow, were employed to conduct predictive analyses across our dataset. Key performance metrics such as accuracy, F1 score, precision, recall, and the area under the receiver operating characteristics curve (AUC) were computed to both train and optimize these models. Results: The Random Forest model achieved an accuracy of 67.5%, with a ROC AUC of 0.67, outperforming the Gradient Boosting and Sequential models, which registered accuracies of 66.3% and 64.5%, respectively. Our results highlighted key predictive factors such as intraocular pressure, family history, and body mass index, substantiating their roles in glaucoma risk assessment. Conclusions: This study demonstrates the potential of utilizing readily available clinical, lifestyle, and demographic data from EHRs for glaucoma detection through deep learning models. While our model, using EHR data alone, has a lower accuracy compared to those incorporating imaging data, it still offers a promising avenue for early glaucoma risk assessment in primary care settings. The observed disparities in model performance and feature significance show the importance of tailoring detection strategies to individual patient characteristics, potentially leading to more effective and personalized glaucoma screening and intervention.

## 1. Introduction

Glaucoma, a leading cause of irreversible blindness, is primarily linked to elevated intraocular pressure (IOP), the only currently modifiable risk factor known to slow the progression of vision impairment [1,2,3,4,5,6,7,8,9,10,11,12,13,14,15,16]. It has been reported that glaucoma will impact roughly 112 million individuals worldwide by 2040 [17]. The American Glaucoma Society notes that while 2.7 million Americans live with glaucoma, a concerning 50% remain unaware of their condition [18]. Also, the disease significantly reduces the quality of life and imposes a considerable economic strain [17], with treatment and healthcare expenses in the U.S. reaching approximately $2.5 billion annually [19]. Detecting glaucoma during its initial stages remains a complex challenge [20,21] due to varying symptoms and rates of disease advancement among individuals [22]. This often results in delayed diagnosis, allowing the disease to progress unchecked until significant visual loss ensues. Consequently, enhancing early glaucoma detection through innovative screening and diagnostic innovation is vital for initiating prompt treatment strategies to lessen vision loss [23,24,25,26].

From the viewpoint of health economics, it is argued that universal glaucoma screening may not be economically viable; however, focusing on individuals at high risk could prove to be more cost-effective [27]. Early detection of high-risk patients within these targeted groups could lead to a more efficient use of healthcare resources, enabling cost-saving management strategies while reducing unnecessary procedures and tests for those at lower risk. This approach has gained particular significance considering the anticipated sharp rise in glaucoma cases due to an aging population, a trend that is unlikely to be paralleled by an increase in the number of healthcare professionals specialized in glaucoma care [28].

Numerous factors have been identified as contributing to the risk of glaucoma development [29]. Currently, a glaucoma risk calculator exists to estimate patients’ risk of developing glaucoma, but it requires many pieces of ocular information (i.e., IOP, optic nerve cup-to-disc ratio, visual field parameters, and corneal thickness) [29], which is available only in the eyecare provider’s office [30,31]. Incorporating the systemic information available from the primary care’s EHR allows us to risk profile patients who have not been seen by eyecare providers [32,33]. Studies have indicated that individuals with systemic health conditions like diabetes experience a 2.8 times higher risk of developing glaucoma compared to those without diabetes [34]. Moreover, vascular issues such as coronary artery diseases have been linked to increased rates of glaucoma, suggesting they could act as indicators for the disease’s progression [35]. Moreover, various population-based studies have identified a correlation between high blood pressure and an increased likelihood of glaucoma [35,36,37]. Furthermore, the impact of medical treatments and the utilization of electronic health records (EHR) on the onset of glaucoma have not been fully explored in either prospective or cross-sectional studies, indicating a potential area for further investigation. Establishing a correlation between clinical, lifestyle, and demographic factors and glaucoma cannot be efficiently achieved through traditional cross-sectional studies alone, as these methods are both time-consuming and costly. While several studies have leveraged EHR for glaucoma prediction, none have incorporated the potentially valuable insights offered by lifestyle and demographic data yet [38,39,40,41].

Recent advancements in technology algorithms have significantly enhanced the detection and diagnosis of various diseases, including glaucoma. Deep learning (DL) algorithms, particularly convolutional neural networks (CNNs), have shown great promise in analyzing medical images such as fundus photography and optical coherence tomography (OCT) scans. Studies by Kim et al. [20] and Asaoka et al. [21] demonstrated the efficacy of CNNs in diagnosing glaucoma, achieving accuracies of 83% and 90%, respectively. Also, ensemble learning methods, as utilized by Norozifar et al. [42] and Chai et al. [43], have improved predictive performance by combining multiple classifiers. Hybrid models that integrate deep learning with traditional machine learning algorithms have also been developed to enhance diagnostic accuracy. Explainable AI (XAI) techniques have been employed to make AI models more interpretable and trustworthy.

**Figure 1:**
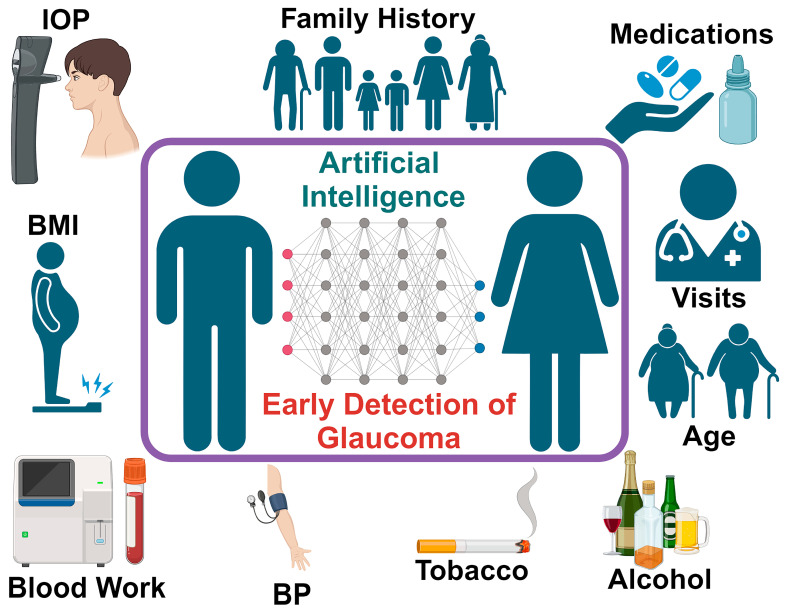
Schematic representation of data utilized in AI-based glaucoma detection, showcasing clinical, lifestyle, and demographic factors.

Deep learning models, a subclass of artificial neural networks, consist of multiple layers of “neurons”, algorithms inspired by biological neural cells. These neurons process inputs from preceding layers, compute an output, and pass it on, functioning collectively within an artificial neural network. These networks process data with the aim of achieving specific outcomes. These have been used for three decades to interpret perimetry data for glaucoma detection [44]. Recent advancements in computational capabilities have enabled the development of deep learning networks with multiple layers capable of handling more intricate data, enhancing performance significantly beyond that of earlier, less complex networks. The application of machine learning for glaucoma diagnosis, particularly through the classification of medical imaging like fundus photography [45,46,47,48], visual fields [28,49,50,51,52], and optical coherence tomography (OCT) [53,54,55,56], has since seen widespread use and demonstrated the potential for early detection. Despite these advances, there remains a gap in utilizing a model that integrates clinical, lifestyle, and demographic data for predicting individual glaucoma risk. This study aims to bridge this gap by developing a comprehensive and robust predictive model using advanced machine learning techniques, specifically, Random Forest and Gradient Boosting algorithms, and a sequential model from the TensorFlow library, to analyze these varied data types. Our goal is to provide a tool that enhances early glaucoma detection and supports clinicians in making informed decisions, thereby improving patient outcomes. Figure 1 illustrates the multifaceted approach of our artificial intelligence model for the early detection of glaucoma, highlighting the integration of various clinical, lifestyle, and demographic factors such as IOP, Body Mass Index (BMI), blood work results, blood pressure (BP), family history, medication use, patient visits, age, tobacco usage, and alcohol consumption. This visual encapsulates the model’s comprehensive data inputs, underscoring the holistic analysis employed to predict glaucoma onset.

The methods employed in this study offer several distinct advantages for glaucoma detection. The reliance on EHR data eliminates the need for invasive procedures or specialized tests, making it a patient-friendly approach. Utilizing existing EHR data is more cost-effective than conducting new clinical trials or developing new diagnostic tools. The data used in this study are readily available in primary care settings, potentially enabling wider screening and earlier detection of glaucoma. By incorporating clinical, lifestyle, and demographic data, the model provides a holistic assessment of glaucoma risk, considering various factors that may contribute to the disease. The methodology can be easily adapted to analyze registry data from different EHR systems, enhancing its applicability across diverse healthcare settings. The model’s ability to identify individuals at high risk for glaucoma before significant vision loss occurs is a major advantage, as early intervention can significantly improve patient outcomes. The model’s focus on individual risk factors allows for personalized risk assessment and tailored treatment strategies, potentially improving the effectiveness of glaucoma management. The use of machine learning algorithms allows for the efficient processing of large datasets, making it scalable for population-level screening and risk assessment.

## 2. Materials and Methods

### 2.1. Participants, Feature Selection, and Dataset Preparation

We obtained deidentified clinical data from an EHR database, which encompassed a broad array of attributes including demographic details, minimum and maximum BMI, diastolic and systolic blood pressures, prescribed medications, number of medical diagnoses, tobacco and alcohol usage, IOP, and results from various laboratory tests. The dataset comprised information on 1937 individuals, of whom 1111 were non-glaucoma patients and 826 were diagnosed with glaucoma. To ensure balance and reduce model bias, we randomly selected a subset of 826 non-glaucoma patients to match the number of glaucoma cases. This study received approval from the institutional review board (IRB #00027899) at the Oregon Health and Science University, Portland, OR, and was conducted in strict adherence to the principles set forth in the Declaration of Helsinki. The methodologies employed in this research are adaptable for analyzing registry data from various EHR systems.

The inclusion criteria for the glaucoma cohort are individuals 40 years or older when glaucoma was diagnosed, with an EHR record between 2012 and 2022, and with at least 3 months of follow-up appointment visits. For the control dataset, we compiled a cohort of patients being evaluated for cataracts without a diagnosis of glaucoma. This approach ensures a clear distinction between cases and controls based on documented medical visits and glaucoma status.

**Figure 2:**
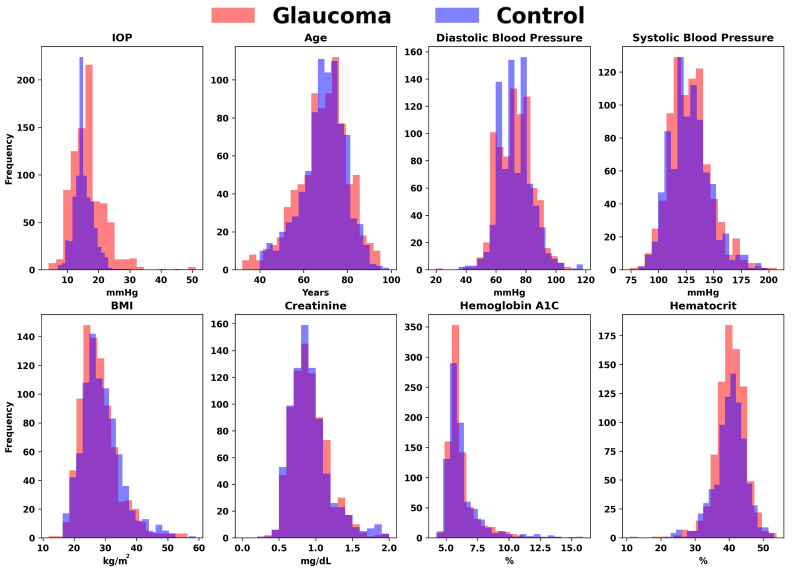
Comparative distributions of key variables for glaucoma versus control groups. The overlaid histograms illustrate the frequency distributions of crucial factors, including Intraocular Pressure (IOP), Age, Diastolic Blood Pressure, Systolic Blood Pressure, BMI, Creatinine, Hemoglobin A1C, and Hematocrit, differentiating between individuals diagnosed with glaucoma and healthy controls.

**Figure 3:**
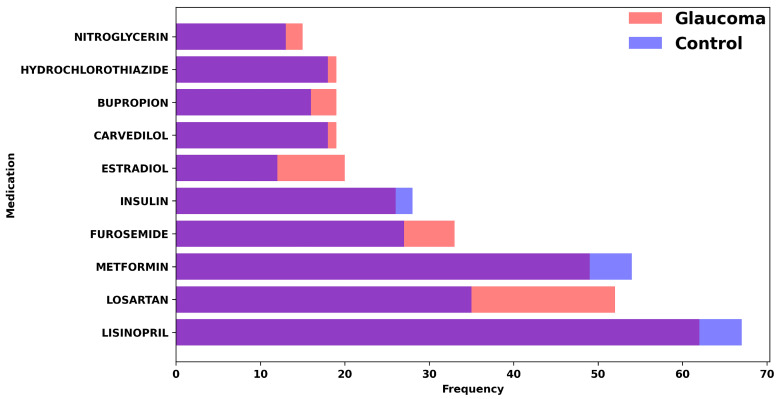
Distribution of medication usage among glaucoma and control groups. This bar chart compares the frequency of various medications, including Lisinopril, Losartan, Metformin, Furosemide, Insulin, Estradiol, Carvedilol, Bupropion, Hydrochlorothiazide, and Nitroglycerin, between individuals diagnosed with glaucoma and control participants.

We started by processing the patient data from Excel sheets into a standardized Comma-Separated Value (CSV) format, with the following factors included from up to 2 years before the glaucoma diagnosis (for glaucoma cohort) and cataract surgery (for control cohort): BMI, Diastolic Blood Pressure, Systolic Blood pressure, Gender, Race, Alcohol Use, Tobacco Use, Age, Family History, IOP, Hemoglobin A1C, Hematocrit, Creatinine Plasma, Past Diagnosis Count, and Medications. Figure 2 represents the quantitative variables used in the dataset, categorized by glaucoma diagnosis. Figure 3 shows the distribution of medication usage among glaucoma and control groups. This bar chart compares the frequency of various medications, including Lisinopril, Losartan, Metformin, Furosemide, Insulin, Estradiol, Carvedilol, Bupropion, Hydrochlorothiazide, and Nitroglycerin, between individuals diagnosed with glaucoma and control participants.

Glaucomatous and non-glaucomatous patients were processed separately, then combined into a single dataset. Due to an emerging separation between the number of glaucomatous and non-glaucomatous patients in the filtered dataset, individuals were systematically undersampled to correct for any bias that could be transferred to the models. All numerical variables, e.g., BMI, blood pressure, age, IOP, hemoglobin A1C, hematocrit, creatinine, and diagnoses, were filtered for outliers and data entry errors. Categorical variables were encoded into discrete numerical values through indexing unique values in the data and creating lookup tables for gender, race, alcohol/tobacco use, and family history. Medication data were condensed to identify unique medications for each patient and split to extract the names of the drugs used. Any individuals without readings for any of these variables, except for medication, were excluded from the dataset. Once the data were preprocessed, the mean and standard deviation of all numerical variables (and any categorical variables encoded to numerical values) were saved and the data were standardized into standard scores in order to be better processed by the TensorFlow library. The extracted mean and standard deviation were later used in the input preprocessing of the application so as to keep user-input data consistent with training and testing data from the dataset. Comparative mean values of clinical parameters between control and glaucoma groups are summarized in Table 1. This table presents the average measurements of IOP, age, diastolic and systolic blood pressure, BMI, creatinine, hemoglobin A1C, and hematocrit for individuals within the control group versus those diagnosed with glaucoma.

### 2.2. Training Models

#### 2.2.1. Sequential Model from Keras Library of TensorFlow

TensorFlow is a versatile and scalable open-source library designed for numerical computation, particularly utilized in machine learning and neural network modeling. It employs dataflow graphs for calculations, optimizing performance with C++ and CUDA for parallel computing, particularly on NVIDIA architectures. TensorFlow supports multiple programming languages, with Python being the most developed and user-friendly interface. This library facilitates the construction and execution phases of machine learning models through an organized computational graph system. In these graphs, nodes represent mathematical operations, while edges denote the multidimensional data arrays (tensors) that traverse the network. TensorFlow provides essential components like layers and activation functions, alongside various loss functions, enabling the effective assembly and training of complex machine learning models, exemplified by applications such as classifying handwritten digits using convolutional neural networks [57]. The TensorFlow model is highly suitable for EHR and early detection of glaucoma due to its ability to handle large datasets and complex data structures typical of EHRs. Its advanced computational capabilities, supported by parallel computing and optimized algorithms, enable the processing and analysis of vast amounts of medical data efficiently. TensorFlow’s flexibility in model architecture allows for the customization of neural networks to identify subtle patterns and indicators relevant to early glaucoma stages, enhancing diagnostic accuracy and patient outcomes.

**Figure 4:**
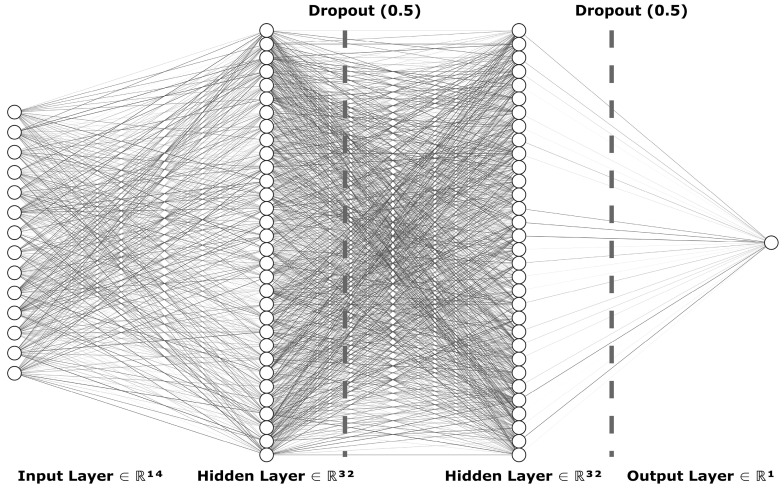
Architecture of the actual deep learning model for glaucoma detection: this diagram illustrates the TensorFlow-based neural network structure, showcasing an input layer with 14 features (ℝ^14^), two hidden layers each with 32 nodes (ℝ^32^) incorporating dropout (0.5) to reduce overfitting, and an output layer (ℝ^1^) for glaucoma risk classification.

To train the multiple layer perceptron model from TensorFlow library, we began by importing the processed data from the CSV file. We used the Pandas Python library for most data transfer between the input files and the machine learning models. The data were then split into training, testing, and validation datasets, by first randomly sampling 80% of the initial data to be used as training, with the remaining becoming test data, and then 30% of the training data to be used for validation so as to prevent model overfitting. The data were then processed into TensorFlow datasets. To accommodate the varied inputs for our TensorFlow model, we employed a Keras Dense Features layer, configuring it primarily for numerical data inputs. This approach streamlined the integration of diverse data types into the model. Medication data were embedded into the feature layer numerically using the TensorFlow library, utilizing a vocabulary list vectorized into a two-dimensional shape. The model itself utilized the aforementioned input layer, leading to a series of two dense layers and an output dense layer. The two hidden layers each consisted of 32 units, using Rectified Linear Unit (ReLU) activation. The output layer used Sigmoid activation. In order to prevent overfitting to the training data, each layer had a quadratic kernel regularizer, followed by a dropout layer with a factor of 0.5. The loss function used for the model was Binary Cross-Entropy, along with the Adam optimizer, targeting accuracy as a main metric. Figure 4 visually depicts the neural network’s structure as designed for our study, illustrating a detailed architecture that includes an input layer capable of processing 14 different features (ℝ^14^), followed by two hidden layers each with 32 nodes (ℝ^32^), and incorporating dropout mechanisms (rate of 0.5) to prevent overfitting. The network culminates in an output layer (ℝ^1^) tailored for binary classification. The diagram was constructed using NN-SVG to clearly present the model’s complexity and functional design. 

**Figure 5:**
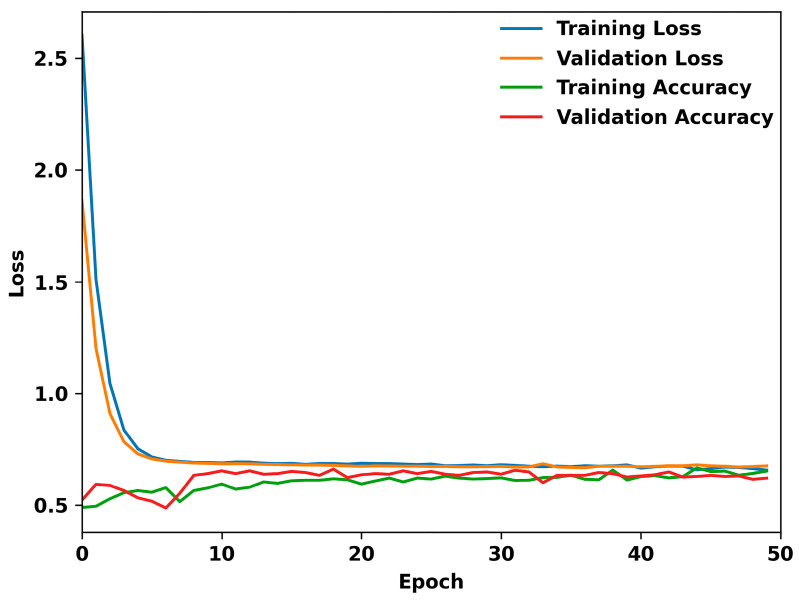
Performance metrics of the TensorFlow model over 50 epochs: this graph illustrates the dynamics of training and validation loss, alongside training and validation accuracy, throughout the model’s learning process.

The model was trained for 25 epochs, and then tested on the test dataset produced earlier. The model was exported using Keras to be included in the application. Figure 5 shows the loss over 50 epochs of example training for the model. Evidently, the model begins to overfit after reaching 30 epochs. The model quickly fits to the data but cannot reduce loss much further. This suggests that there is not enough correlation to continue learning from the data.

**Figure 6:**
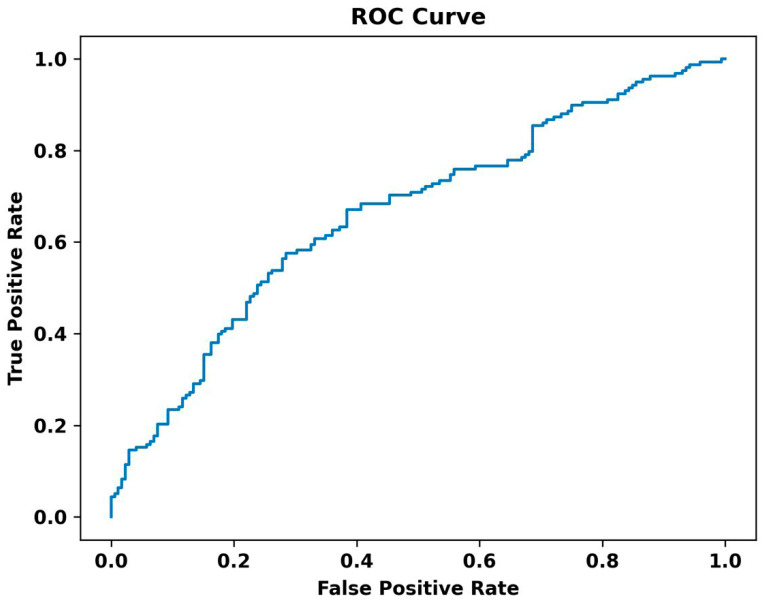
The ROC curve of the TensorFlow model.

The ROC curve of the TensorFlow model is also illustrated in Figure 6. The curve plots the True Positive Rate (Sensitivity) against the False Positive Rate (1-Specificity) at various threshold settings. The area under the ROC curve (AUC) provides a single measure of overall accuracy. In our analysis, the ROC curve demonstrates a moderate classification performance, with an AUC value of 0.645. This indicates that the model performs better than random chance, effectively distinguishing between the classes.

#### 2.2.2. Random Forest and Gradient Boosting

The Random Forest model is a robust machine learning algorithm that operates by constructing multiple decision trees during the training phase and outputting the mode of the classes (for classification tasks) or mean prediction (for regression tasks) of the individual trees. It excels in handling large datasets with higher dimensionality and can manage missing values and maintain accuracy even when a large proportion of the data is missing. Random Forests perform well for a wide range of data types and are particularly noted for their ability to mitigate overfitting, making them highly reliable and versatile for predictive modeling. This ensemble approach increases predictive accuracy and controls overfitting by averaging the results from multiple decision trees constructed on different subsets of the dataset [58,59]. The Random Forest algorithm is particularly suitable for EHR and early detection of glaucoma due to its ability to handle heterogeneous data types, manage missing values, and process complex interactions between variables. This is crucial when working with EHR, which often contains incomplete and varied data. Furthermore, Random Forest’s ensemble approach reduces the risk of overfitting, enhancing the reliability of early glaucoma detection by providing more generalized and robust predictive insights from patient records.

The Gradient Boosting model is a robust machine learning technique that builds predictive models in the form of an ensemble of weak prediction models, typically decision trees. It improves model predictions iteratively by correcting errors from previous models through optimized weight adjustments [60,61]. This approach is highly effective for complex datasets like those found in healthcare, making it particularly valuable for tasks such as the early detection of diseases. Gradient Boosting handles various data types and distributions effectively, making it suitable for nuanced tasks like analyzing EHR for early signs of conditions such as glaucoma by capturing intricate patterns and relationships within the data.

**Figure 7:**
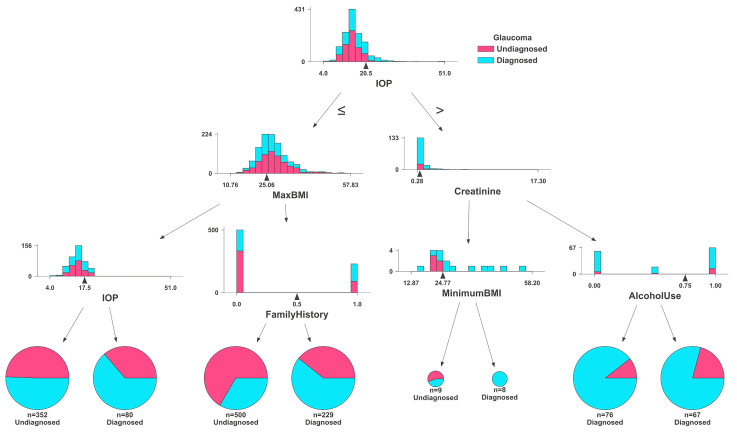
Feature distributions by glaucoma diagnosis status: comparative histograms and pie charts illustrating variations in IOP, BMI, family history, creatinine levels, and alcohol use between diagnosed and undiagnosed glaucoma patients, as analyzed by the Random Forest classifier.

The Random Forest and Gradient Boosting algorithms were both used from the Python library SciKit-Learn. Data were loaded from CSV format in the same way as the TensorFlow model. The Random Forest and Gradient Boosting models did not use medication data as the TensorFlow model did. It was separated into training and test datasets, with 80% of the data being used for training, and the remainder for evaluation. Randomized hyperparameter search was conducted, resulting in optimal hyperparameters of 15 and 17 estimators for the Gradient Boosting and Random Forest algorithms, respectively, and with 3 being used as the maximum depth for each. The models were fitted to the data and evaluated using the test data. Figure 7 shows the graph of one decision tree used in the Random Classifier. This figure, created with the DTreeViz Python library, demonstrates a single estimator (or decision tree) from the ensemble, which votes on the final outcome of the model. Individuals are first classified by IOP in this tree, and then processed into smaller “bags”, until reaching a conclusion. The more homogenous a final bag is, the more effective the classification. This includes histograms for IOP, maximum BMI, creatinine levels, and minimum BMI, alongside pie charts depicting distributions of family history and alcohol use. Each histogram and pie chart delineate the frequency or proportion of these variables within the glaucoma and control cohorts, showcasing differences and similarities that aid in the analysis and understanding of the risk factors associated with glaucoma.

## 3. Results

**Figure 8:**
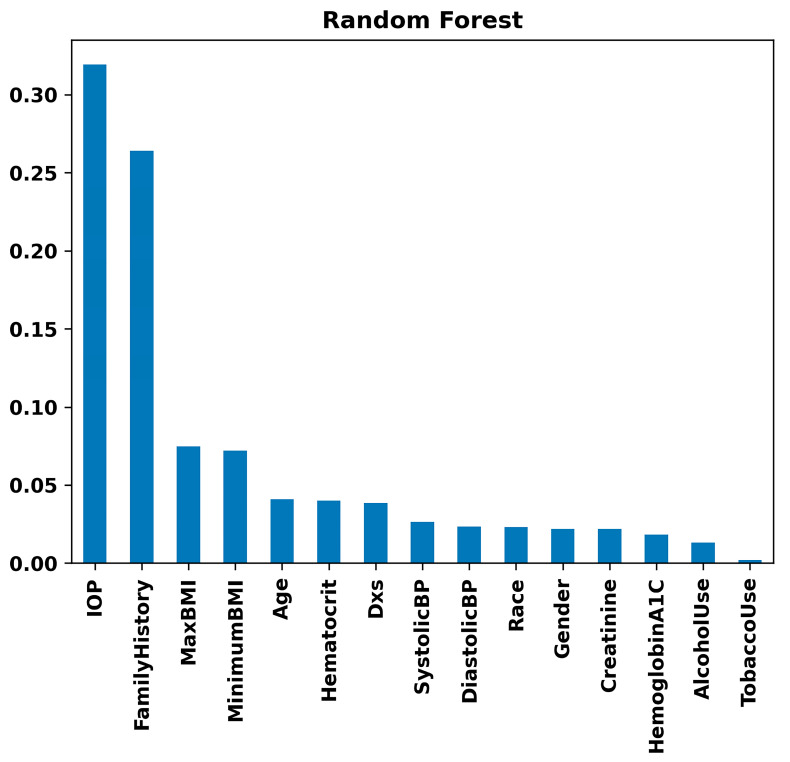
Feature importance rankings from the Random Forest model: bar chart displaying the relative importance of clinical, lifestyle, and demographic variables, including IOP, family history, BMI, and others, in predicting glaucoma.

**Figure 9:**
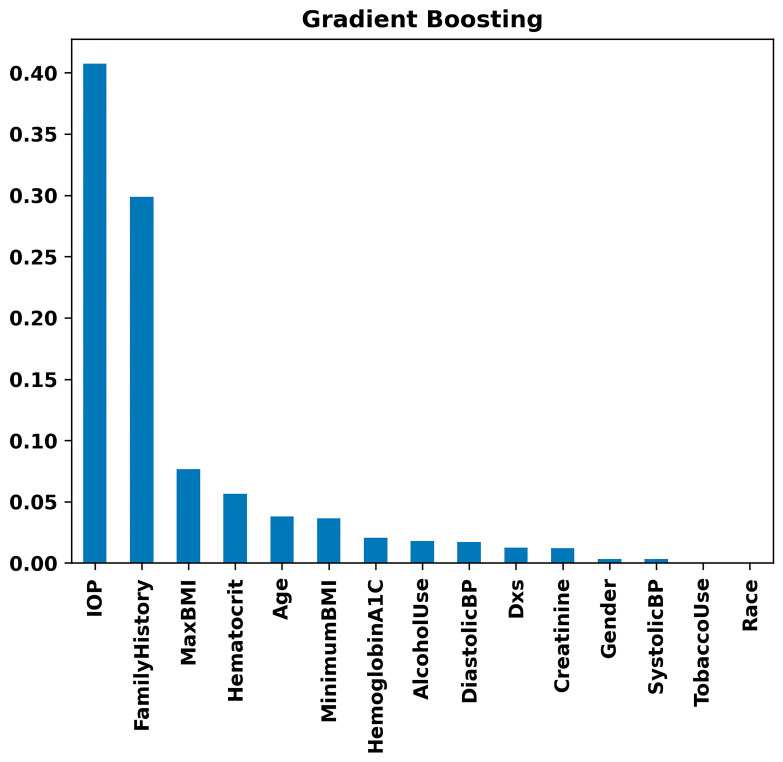
Feature importance rankings from the Gradient Boosting model: bar chart displaying the relative importance of clinical, lifestyle, and demographic variables, including IOP, family history, BMI, and others, in predicting glaucoma.

Figure 8 highlights IOP and Family History as substantial risk factors using the Random Forest model. Maximum BMI and Minimum BMI follow, showing moderate importance. Variables such as Age and Hematocrit show lower importance but are still relevant. In contrast, parameters like Systolic Blood Pressure, Diastolic Blood Pressure, Race, Gender, Creatinine, Hemoglobin A1C, Alcohol Use, and Tobacco Use demonstrate minimal impact on the model’s decision-making process for glaucoma prediction. This suggests that while IOP and Family History are critical in assessing the risk of glaucoma, other collected variables have less predictive value in this specific model context. The same results were observed with the Gradient Boosting model (Figure 9). That is, in the Gradient Boosting model, IOP emerges as the most significant feature in predicting glaucoma, with Family History also showing substantial importance. These are followed by Maximum BMI and Hematocrit, indicating a moderate level of significance. Other variables such as Age, Minimum BMI, and Hemoglobin A1C exhibit lower levels of importance. Particularly, factors like Alcohol Use, Diastolic Blood Pressure, Number of Visits (Dxs), Creatinine, Gender, Systolic Blood Pressure, Tobacco Use, and Race are demonstrated to have minimal influence on the model’s predictive capabilities regarding glaucoma. This suggests that the Gradient Boosting model prioritizes physiological and hereditary factors over lifestyle choices and demographic characteristics in glaucoma risk assessment. 

**Figure 10:**
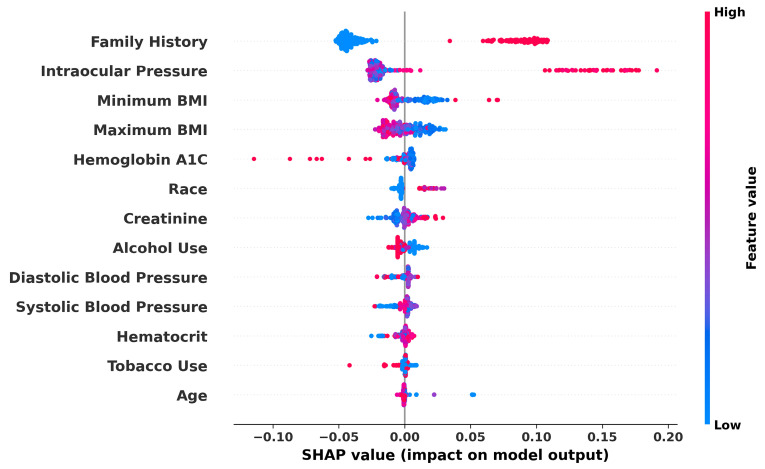
SHAP summary plot demonstrating the impact of various features on glaucoma prediction: the graph visualizes the SHAP values for each feature used in the Random Forest model, indicating the influence of factors like family history, IOP, and BMI on model output. Higher feature values push the model output from Low (blue) to High (red) risk of glaucoma.

The directional impact of each feature can be specifically visualized with SHAP values as shown in Figure 10, which is not compatible with the Gradient Boosting implementation used from SciKit-Learn. The graph displays impact data from the Random Forest model. The graph reveals some expected and some unexpected correlations. For example, IOP and Family History have a high positive correlation with estimated glaucoma risk, while BMI appears to have a weak negative correlation. Race and Gender are not useful to interpret from this graph, as the mapped values are arbitrary, but because Tobacco and Alcohol Use were mapped so that a higher value relates to a higher use, we can see that Tobacco Use is very weakly negatively correlated with predicted glaucoma risk. Systolic Blood Pressure is positively correlated, but diastolic does not appear to hold the same correlation, as most data points are centered around the origin. Overall, our analysis delineates the profound utility of machine learning in refining glaucoma detection methods, specifically highlighting the critical roles of genetic predisposition, physiological measurements, and demographic factors in influencing disease risk and model predictions.

**Figure 11:**
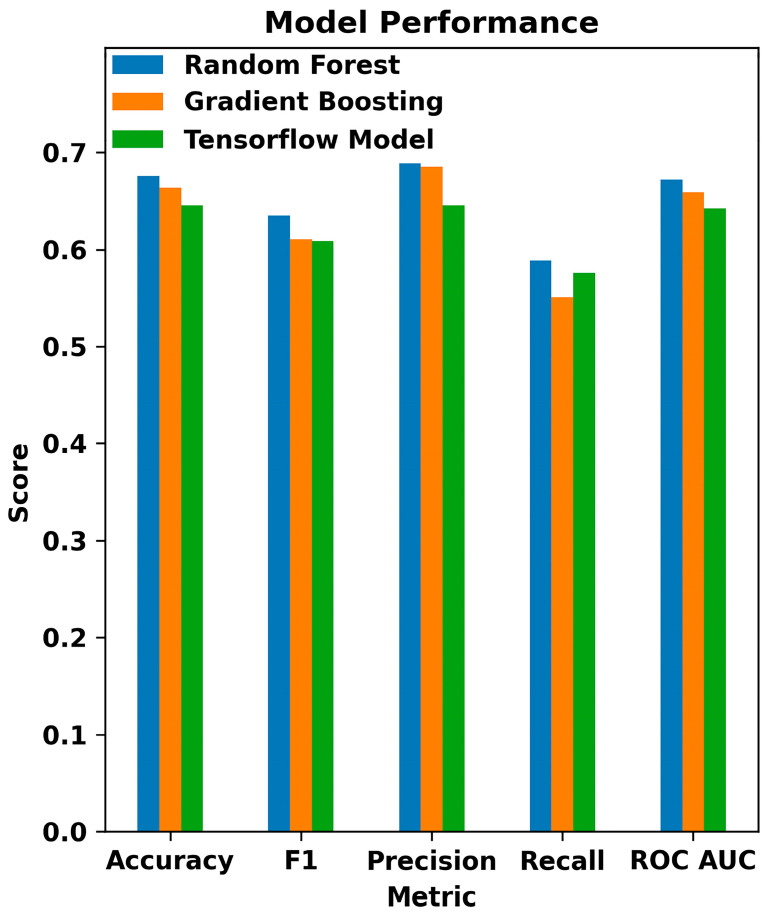
Comparative performance metrics of machine learning models in glaucoma detection: this bar chart displays the evaluation of Random Forest, Gradient Boosting, and TensorFlow models across various classification metrics, including Accuracy, F1 Score, Precision, Recall, and ROC AUC.

The three model types used were evaluated based on the models’ accuracy, F1 score, precision, recall, and Area Under the Curve (AUC) as displayed in Figure 11. This graph shows the comparisons between the three models. The Random Forest classifier outperforms the other two, though with lower precision than Gradient Boosting. Interestingly, the Sequential model from the Keras library of TensorFlow performed the worst on all metrics except recall. This may be due to the relatively low population size of 1652 individuals used in training, as decision tree classifiers tend to perform better with small data sets. The sparse nature of medication data may also reduce some metrics, as only a minority of individuals in the dataset had recorded data on their medication history. If the model was retrained on a larger and more complete dataset, we could expect it to outperform the decision tree-based classifiers, especially with more complex factors like medication data.

Supplementary Figure S1 illustrates an interface snapshot of our glaucoma risk assessment tool, showcasing the initial user inputs such as BMI, Blood Pressure, Intraocular Pressure, Age, and other clinical, lifestyle, and demographic details. The figure also displays risk assessments generated by the Random Forest, Gradient Boosting, and comprehensive AI (TensorFlow) models based on the provided data, highlighting the tool’s capacity to evaluate glaucoma risk with varying levels of confidence across different machine learning approaches.

## 4. Discussion

Glaucoma, a chronic condition leading to irreversible blindness, presents significant challenges in early diagnosis. However, recent advancements in computer-aided techniques create new opportunities for glaucoma screening and diagnosis. This study leverages sophisticated machine learning algorithms, namely Random Forest, Gradient Boosting, and the Sequential model from the TensorFlow library. We found that a combination of clinical, lifestyle, and demographic information in the EHR can help predict the diagnosis of glaucoma. Our findings highlight the potential of AI in assisting the diagnosis and screening of glaucoma, with the hope of improving patient outcomes and reducing vision loss.

Our analysis revealed that IOP, Family History, and BMI stand out as principal risk factors for glaucoma (Figure 7, Figure 8 and Figure 9), aligning with the existing literature [56,62,63,64,65]. IOP and family history are well-known risk factors for glaucoma, but existing research presents conflicting findings between BMI and glaucoma, where some studies report no significant association [66], while others suggest a positive correlation [67] or indicate a negative relationship [68]. Aligning with the findings of the most extensive study to date [67], our data suggest a positive correlation, indicating that a higher BMI may be associated with an increased risk of glaucoma. It is likely that the relationship between BMI and glaucoma is non-linear and complex. For example, a higher BMI is associated with increased IOP, while a lower BMI is associated with higher translaminar pressure across the optic nerve. Machine learning methods may be better at exploring complex relationships compared to conventional biostatistics that use mean to analyze data. 

We found several laboratory tests to be important for the risk prediction model. Hemoglobin A1C, a blood test that indicates diabetes control, was found to be a risk factor for glaucoma (Figure 9). Diabetes has been associated with glaucoma, although the relationship is again complex and likely non-linear. Diabetes likely contributes to endothelial dysfunction, leading to decreased perfusion to the optic nerve. However, certain diabetes medications have neuroprotective effects and have been found to decrease the risk of glaucoma. While this is still preliminary, our study highlights the importance of further investigation to explore whether managing hemoglobin A1C levels could have a reducing effect on glaucoma diagnosis. Creatinine level indicates renal function, and our analysis suggests lower values correlate with a higher risk for glaucoma and vice versa. This may seem counter-intuitive at first that better renal functions would be a risk for glaucoma, but again complex relationships exist in these clinical conditions. Patients with renal insufficiency may be placed on medications that have neuroprotective effects, as diabetes-related nephropathy is a major cause for renal disease. These findings are the first step in identifying and exploring broader vascular and systemic health conditions in glaucoma diagnosis and underscore the potential interconnections between systemic health and ocular conditions.

Numerous studies have highlighted various risk factors contributing to the onset and progression of glaucoma, with age consistently recognized as a predominant non-modifiable factor [69]. However, delineating the impact of preventable risk factors separate from aging remains complex due to their interrelated nature [70]. Since glaucoma is an aging disease [71,72,73] and, in our study, age did not emerge as a significant differentiator, likely because both our glaucoma and control groups were composed predominantly of older individuals. Remarkably, genetic factors, particularly within the African American population, have been identified as significant, with research pointing to specific genetic variations associated with increased susceptibility to primary open-angle glaucoma [74,75,76,77]. Furthermore, while obesity, hypertension, cataracts, atherosclerosis, and type 2 diabetes have all been cited as potential predictors for glaucoma [22], our analysis did not prioritize hypertension as a leading risk factor, diverging from previous findings where hypertension showed a strong association with glaucoma. This discrepancy emphasizes the complexity of glaucoma’s risk factors and underscores the need for tailored approaches in glaucoma screening and prevention, particularly in genetically predisposed populations (Figure 10).

Our analysis considered the impact of various medications on glaucoma outcomes. In line with studies like [78], we found that the inclusion of medications such as metformin had a notable influence on the model’s predictions. Conversely, the exclusion of most oral medications, which have minimal effectiveness against glaucoma, helped to refine our model and reduce potential confounding effects. These findings underscore the importance of carefully selecting relevant clinical variables to enhance the model’s accuracy and reliability.

The variability in glaucoma definitions and testing equipment, as highlighted by [79], underscores the importance of developing standardized protocols for AI applications in glaucoma care. This variability can lead to inconsistencies in diagnosis and treatment, which our model aims to address through rigorous validation and adaptation to different clinical settings. Future work should focus on collaborating with a broad range of clinical practices to refine these standards and enhance the model’s generalizability and reliability.

### Limitations

First, inherent biases in the dataset can inadvertently be absorbed by the model. For instance, previous research indicates that African American individuals are at a higher risk for glaucoma. However, in this study, due to the predominantly white demographic of Oregon State, we faced limitations in accessing a sufficient number of African American participants [75,76,77,78]. Consequently, our dataset lacks substantial data on African American individuals, which may affect the accuracy of our model’s race-based predictions. Enhancing the model’s accuracy could be achievable by integrating a larger and more diverse dataset, including imaging data, to create a more generalizable model of glaucoma detection [80].

Second, despite progress in demystifying the decision-making processes of deep learning models, description continues to be a major challenge. Clinicians are more likely to trust models that are explainable. We used Shapley values to explain our input variables, but this is not possible with all models. This underscores the critical need for transparency in the application of AI to medical decision-making.

Third, most deep learning models offer only a probability of diagnosis rather than absolute certainty. Unlike human experts who might err on complex cases, deep learning models can falter on simpler cases, sometimes assigning high likelihoods to incorrect decisions. This underscores the unique limitations of AI compared to human judgment in medical diagnostics.

Fourth, EHR studies rely on the accuracy of diagnosis labels. Since 50% of individuals with glaucoma are not aware of their disease, there is a potential for a high false negative rate in the control group. We decreased this likelihood by using a control cohort with a complete eye exam by an ophthalmologist for potential cataract evaluation, which decreases the likelihood of unreported glaucoma. However, it is still possible that diagnosis codes are inaccurately assigned. In the future, developing an algorithm that uses multiple EHR data (i.e., glaucoma medications, exam findings) can help verify the diagnosis.

Fifth, the accuracy values obtained in our study are lower compared to some other studies [20,21,76]. In our study, the Random Forest model achieved an accuracy of 67.5%, while the Gradient Boosting and multiple layer perceptron models achieved accuracies of 66.3% and 64.5%, respectively. These values, although lower than some reported in the literature, are consistent with the inherent complexity and heterogeneity of the data used. The studies mentioned primarily used high-quality imaging data such as fundus photography and OCT images, which provide detailed structural information about the eye. In contrast, our study relied on EHR, which includes a broader array of clinical, lifestyle, and demographic data. These data types can introduce more variability and noise, potentially impacting model accuracy. The dataset size and population diversity also play critical roles in model performance. Our dataset included 1652 participants, while some of the referenced studies used larger and more homogenous datasets, contributing to higher accuracy. The preprocessing techniques and feature selection criteria can significantly affect model performance. While we included a wide range of features from EHR, differences in feature engineering and selection can lead to variations in accuracy. It is also important to note that our study’s primary goal was not to achieve the highest possible accuracy but rather to explore the potential of using EHR data for early glaucoma detection. The results, while not as high as some imaging-based studies, still demonstrate the feasibility and potential value of this approach. Future studies incorporating larger and more diverse datasets, as well as additional data modalities like imaging, could potentially improve the accuracy and clinical utility of such predictive models.

Finally, in this study, distinctions among primary open-angle glaucoma, high-tension glaucoma, and normal-tension glaucoma were not delineated. However, we anticipate that incorporating OCT images in future iterations of our model will enable us to include and differentiate between these specific types of glaucoma, enhancing the model’s diagnostic capability and precision.

## 5. Conclusions

Deep learning shows promise for glaucoma diagnosis, especially by identifying the disease through EHR, which could lead to cost-effective screening methods. Our research shows the potential that machine learning methods, such as Random Forest, Gradient Boosting, and the Sequential model from the TensorFlow library, can have on the early detection of glaucoma. By harnessing a wide-ranging dataset incorporating clinical, lifestyle, and demographic factors, we demonstrated the role of including a variety of data elements to improve diagnostic precision in the model. Also, our study corroborates the importance of factors like IOP, BMI, and Family History as crucial predictors, as well as highlights other factors for future research to expand our understanding of pathophysiology of glaucoma. The integration of machine learning into ophthalmology has the potential for more timely diagnoses and the prevention of vision loss. 

The path ahead requires ongoing research to refine these technologies, improve their transparency, and ensure their ethical use in healthcare settings. Looking to the future, the synergy between artificial intelligence and clinical practice promises to enhance glaucoma management and the detection and treatment of a broader range of eye conditions. Encouraging collaboration between data scientists and medical professionals will be crucial in harnessing AI’s full potential to advance preventive ophthalmology and elevate patient care standards. 

## Figures and Tables

**Table 1:** Comparative mean values of clinical parameters between control and glaucoma groups. This table presents the average measurements of intraocular pressure (IOP), age, diastolic and systolic blood pressure, body mass index (BMI), creatinine, hemoglobin A1C, and hematocrit for individuals within the control group versus those diagnosed with glaucoma.

Parameters	Control	Glaucoma
IOP (mm Hg)	15.18	16.68
Age (year-old)	69.25	68.79
Diastolic blood pressure (mm Hg)	73.2	72.8
Systolic blood pressure (mm Hg)	128.2	128.49
Body mass index (kg/m^2^)	28.69	27.89
Creatinine (mg/dL)	0.99	1.04
Hemoglobin A1C (%)	6.27	6.21
Hematocrit (%)	40.28	40.4

## Data Availability

The raw/processed data required to reproduce these findings cannot be shared at this time as the data are part of an ongoing study.

## Supplementary Materials

The following supporting information can be downloaded at: https://www.mdpi.com/article/10.3390/bioengineering11060577/s1, Figure S1: An interface snapshot of our glaucoma risk assessment tool, showcasing the initial user inputs such as BMI, Blood Pressure, Intraocular Pressure, Age, and other clinical, lifestyle, and demographic details. The figure also displays risk assessments generated by the Random Forest, Gradient Boosting, and comprehensive AI models based on the provided data, highlighting the tool’s capacity to evaluate glaucoma risk with varying levels of confidence across different machine learning approaches.
