# Supplementary figures and images for: Integrating Deep Learning with Electronic Health Records for Early Glaucoma Detection: A Multi-Dimensional Machine Learning Approach
Source: Bioengineering (Basel). 2024 Jun 7;11(6):577. doi: 10.3390/bioengineering11060577 (PMC11200568; doi:10.3390/bioengineering11060577)

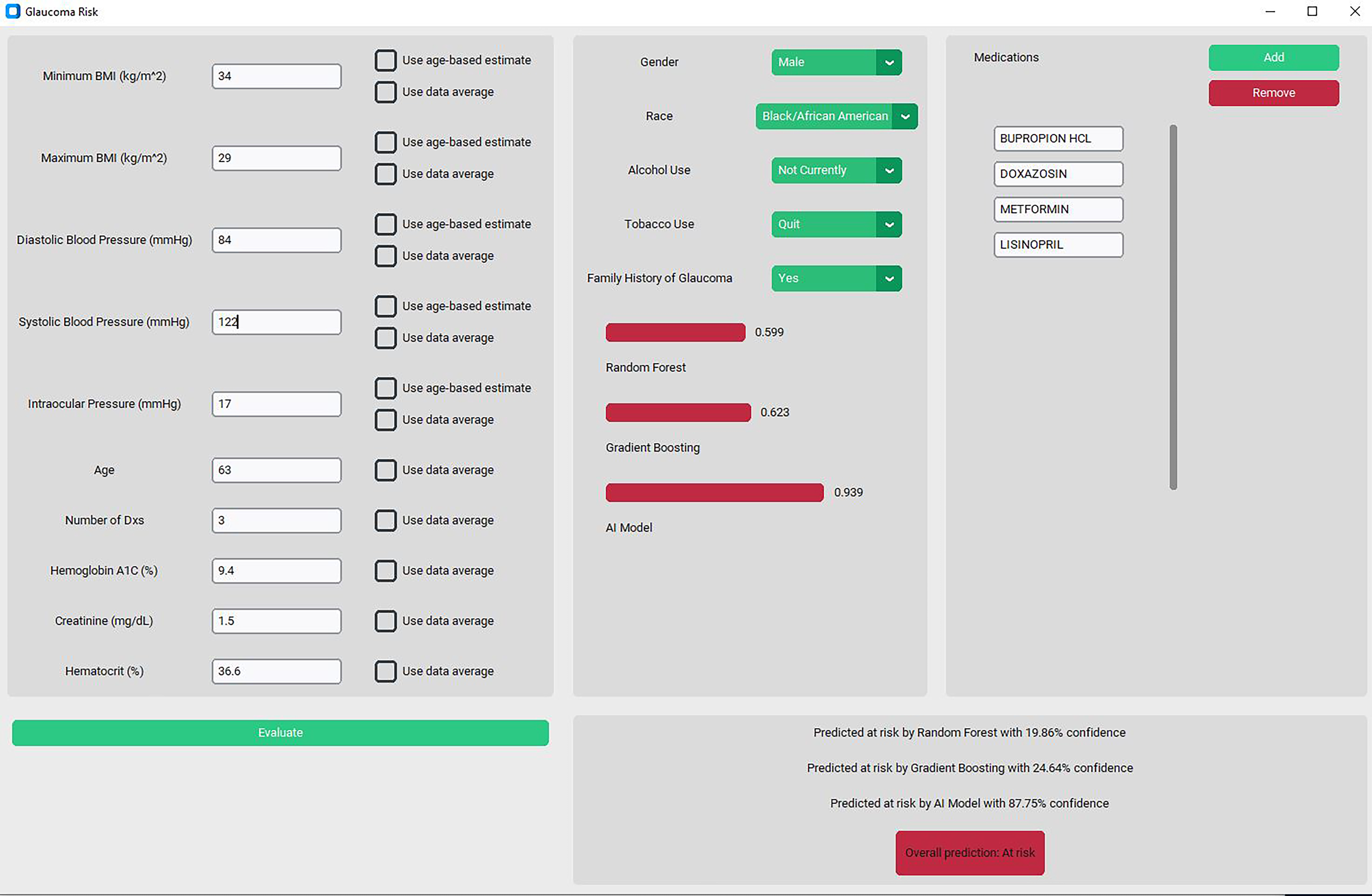

Supplement: Supplementary file 1 [file bioengineering-11-00577-s001.zip › bioengineering-3037411-supplementary.tif]
